# Revisiting Street Intersections Using Slot-Based Systems

**DOI:** 10.1371/journal.pone.0149607

**Published:** 2016-03-16

**Authors:** Remi Tachet, Paolo Santi, Stanislav Sobolevsky, Luis Ignacio Reyes-Castro, Emilio Frazzoli, Dirk Helbing, Carlo Ratti

**Affiliations:** 1 Senseable City Laboratory, Massachusetts Institute of Technology, 77 Massachusetts Avenue, Cambridge, MA 02139, United States of America; 2 Istituto di Informatica e Telematica del CNR, Pisa, Italy; 3 Laboratory for Information and Decision Systems, Massachusetts Institute of Technology, 77 Massachusetts Avenue, Cambridge, MA 02139, United States of America; 4 ETH Zurich, Computational Social Science, Clausiusstrasse 50, CH-8092, Zurich, SWITZERLAND; Beihang University, CHINA

## Abstract

Since their appearance at the end of the 19th century, traffic lights have been the primary mode of granting access to road intersections. Today, this centuries-old technology is challenged by advances in intelligent transportation, which are opening the way to new solutions built upon slot-based systems similar to those commonly used in aerial traffic: what we call Slot-based Intersections (SIs). Despite simulation-based evidence of the potential benefits of SIs, a comprehensive, analytical framework to compare their relative performance with traffic lights is still lacking. Here, we develop such a framework. We approach the problem in a novel way, by generalizing classical queuing theory. Having defined safety conditions, we characterize capacity and delay of SIs. In the 2-road crossing configuration, we provide a capacity-optimal SI management system. For arbitrary intersection configurations, near-optimal solutions are developed. Results theoretically show that transitioning from a traffic light system to SI has the potential of doubling capacity and significantly reducing delays. This suggests a reduction of non-linear dynamics induced by intersection bottlenecks, with positive impact on the road network. Such findings can provide transportation engineers and planners with crucial insights as they prepare to manage the transition towards a more intelligent transportation infrastructure in cities.

## Introduction

Understanding the dynamics of networks composed of a large number of interacting elements is an important, but highly complicated, scientific challenge with prominent real-world applications, such as the study of traffic flows in cities [1, 2]. The latter is an arduous problem characterized by many elements (vehicles, traffic lights) that are highly constrained in space and time. Constraints apply not only to the amount of vehicles on a given road section, but are also generated by conflicts of usage at designated zones (intersections). The combination of the above factors gives rise to highly non-linear and difficult-to-predict dynamics. This explains why traffic can rapidly deteriorate in cities, resulting in widespread congestion and immense societal and environmental costs [3].

Intersections are the physical place where access to a common resource (the intersection area) must be coordinated between vehicles with incompatible paths. As such, they are natural bottlenecks and play a key role in the dynamics of the network. Coordination of vehicles is achieved by means of a switching process, the purpose of which is to resolve conflicts between incompatible flows, while optimizing some system performance metric. The state-of-the-art embodiment of this switching process is the well-known traffic light [4], which has been in operation in its present form for approximately 150 years.

Traffic lights are operated according to a phased switching process, which is often periodical: a relatively long time period *T* called cycle is divided into a number of “phases” *P*_1_, *P*_2_, … of duration *t*_1_, *t*_2_, … , and, during each phase, only a number of non-conflicting flows is given access to the intersection. Transition from one phase to the next is not instantaneous, but requires a “setup phase” (amber light) which typically lasts between 5 and 8 seconds [5]. Since the intersection operates in highly sub-optimal conditions during the “setup phase”, there is an inherent tradeoff between ‘delay’ and ‘capacity’ when operating traffic lights. ‘Delay’ is generally defined as the difference between the time needed by a vehicle to complete a travel in free flow conditions, and the travel time needed in reality [4]. ‘Capacity’ is equal to the maximum vehicle arrival rate before delays stop being bounded in time and grow to infinity. The tradeoff between ‘delay’ and ‘capacity’ can be easily understood: short phase durations and frequent phase transitions reduce the average delay experienced by vehicles in crossing the intersection. However, frequent phase transitions require a relatively high number of “setup phases”, during which the intersection throughput is considerably reduced.

New information and control systems are paving the way to novel traffic management approaches. For example, vehicles might communicate with roadside infrastructure and other vehicles to produce better coordinated flows [6]. Furthermore, autonomous driving is starting to enable the careful control of vehicle trajectories and the synchronization of their arrival times at the intersections [7]. The underlying principle resembles slot-based control systems used for the management of planes at airports. In short: *i*) time slots for safely accessing the intersection area are assigned to individual vehicles, based on a carefully designed scheduling algorithm; and *ii*) vehicles control their speed to reach the intersection at the beginning of the assigned time slot.

At a first glance, Slot-based Intersections (SIs) are subject to the same inherent tradeoff between delay and capacity discussed above for traffic light systems. A first-come-first-serve approach could be realized by accelerating or decelerating vehicles such that they arrive at the intersection when gaps in the conflicting traffic flows have been created for them. However, a one-by-one service policy is not efficient at high vehicle arrival rates. Then, forming platoons of vehicles and serving all vehicles in the platoon before giving way to a conflicting flow is more efficient from a capacity point of view. This raises the question: how efficient slot-based control systems would be as compared to traffic-light-based controls? This is the central question of this article.

Different types of SIs have recently been proposed [6–10] and, based on simulation results, it has been suggested that they might be more efficient than traditional traffic lights [6–8]. Yet, a theoretical and comprehensive framework to assess their performance and compare it with traffic lights is still lacking. Without it, it is not possible to determine whether, in a non-distant future where SIs are technically feasible, traffic light intersection management systems will still be the preferred solution for intersection management. Answering such question is urgent, as the transportation infrastructure that is being built today will be in operation for several decades and will most likely experience the transition to more intelligent, autonomous transportation [11].

**Fig 1:**
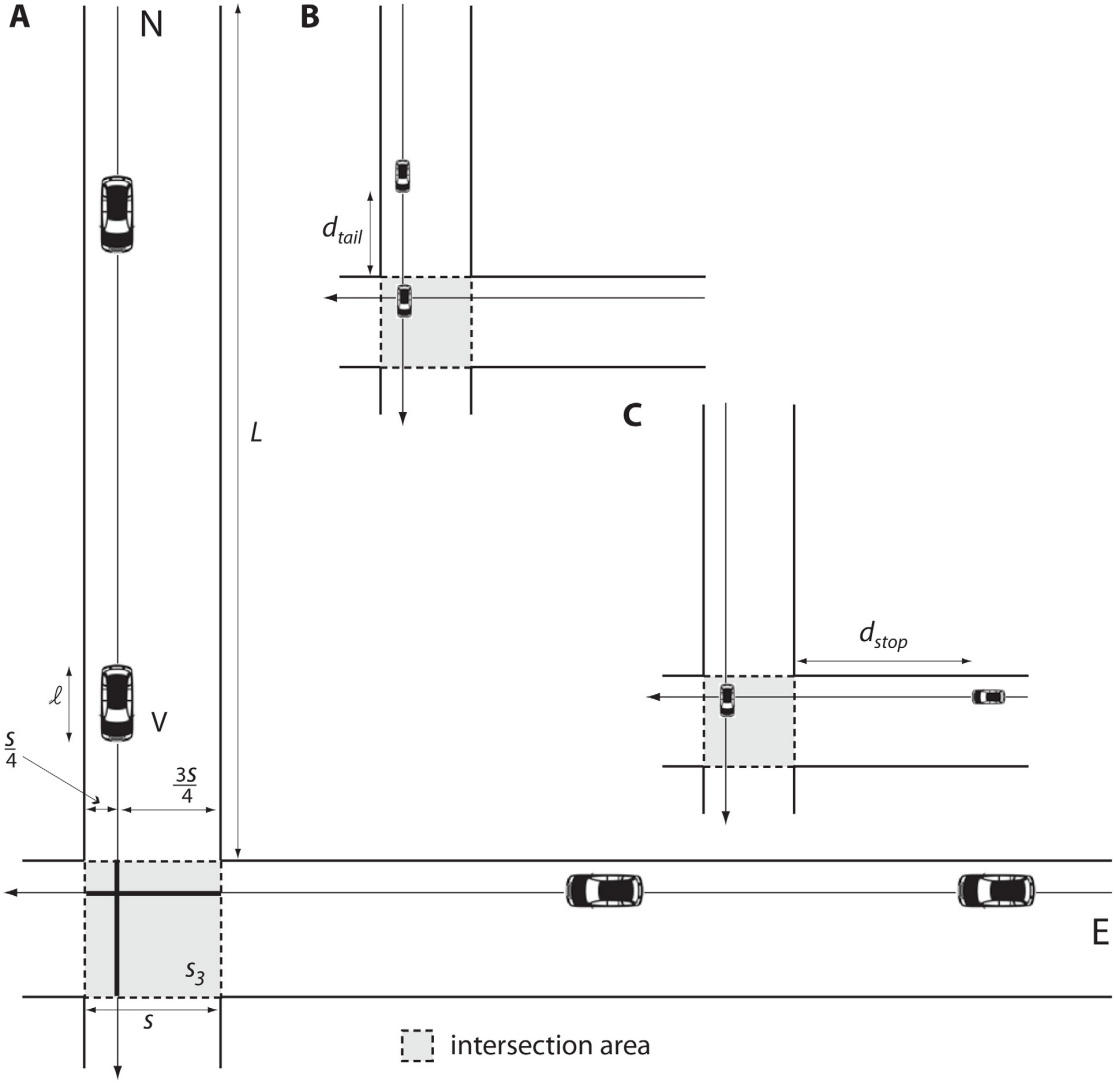
Road intersection scenario. (A) The road system is composed of two single-lane roads of length *L*, crossing at an intersection. The square intersection area of side *s* is shaded. Vehicles, each of length ℓ, enter the system at the beginning of the North road if they belong to the *N* flow, or at the beginning of the East road if they belong to the *E* flow. To model a worst-case situation, vehicles approaching the intersection from different flows are assumed to have a conflicting trajectory: e.g., going straight. A vehicle’s intersection access time is defined as the time at which the head of the vehicle enters the intersection area. (B) Safety requirements dictate that two vehicles consecutively accessing the intersection and belonging to the same flow must be separated by tailgate distance *d*_*tail*_. (C) If the two consecutive vehicles belong to different flows, they must be separated by vehicle stopping distance *d*_*stop*_, which is larger than *d*_*tail*_ for practical values of the system parameters. The tailgate and vehicle stopping distance are formally defined in S1 Supporting Information.

The first step is identifying the key performance parameters to be used for comparison. We postulate that the first parameter to be considered should be safety, as one cannot accept that intersection control purely based on efficiency metrics might put people’s lives at risk. We address safety at the level of both a single vehicle traveling along a trajectory, and of multiple vehicles traveling along potentially conflicting trajectories (see SI for details). Intersection access is granted based on two notions of safety distance: tailgate distance *d*_*tail*_ and stopping distance *d*_*stop*_ (Fig 1B and 1C and S1 Supporting Information). All safety parameters being equal, we then compare different systems using the classic metrics of delay and capacity [4].

We use queuing theory [12] to estimate the performance of different intersection management systems. In particular, we show how to extend classical queuing theory and formally characterize the delay and capacity of a SI—something that has not been done to date and that allows a mathematically accurate comparison with traffic light systems.

## Results

The road system analyzed here is composed of two vehicle flows crossing at a common intersection area (Fig 1A). Generalization to an arbitrary number of roads and lanes crossing at an intersection is reported in the S1 Supporting Information. We consider two flows of vehicles entering the system at the beginning of their respective roads (Fig 1A): one from North to South (*N* flow), and one from East to West (*E* flow). Vehicles enter the system according to two independent Poisson processes of fixed rates *λ*_*N*_ and *λ*_*E*_. Since vehicle flows cross in the intersection area (Fig 1A), intersection access times should then be optimally scheduled in order to maximize capacity, minimize average delay, or obtain a tradeoff between the two performance metrics. Since *d*_*tail*_ < *d*_*stop*_ (Fig 1), grouping together consecutive vehicles from the same flow (say, the *N* flow) increases system capacity. However, incoming vehicles from the *E* flow experience relatively higher delays due to yielding intersection access to the group of vehicles in the *N* flow. This leads to a tradeoff between capacity and delay, which is addressed in the following by introducing two intersection management strategies. The first strategy is designed to privilege fairness between vehicle flows over capacity, and it is hence named FAIR. Vehicle requests are served in a first-come-first-serve fashion—see S1 Supporting Information for details. Requests are labeled with either an *N* or *E* label depending on the flow they belong to. The vehicle service time *T* depends on its flow and on the flow of the following vehicle: if both are the same, we have *T* = *T*_1_, otherwise *T* = *T*_2_ > *T*_1_. The specific values of *T*_1_ and *T*_2_ are determined by safety considerations, as well as by the geometry of the intersection (S1 Supporting Information). The service time *T* is thus a discrete random variable with possible values *T*_1_ and *T*_2_, and the system at hand becomes an instance of M/G/1 queue in the classic theory [12]. Capacity and stationary delays of the slot-based intersection can be readily derived (S1 Supporting Information).

**Fig 2:**
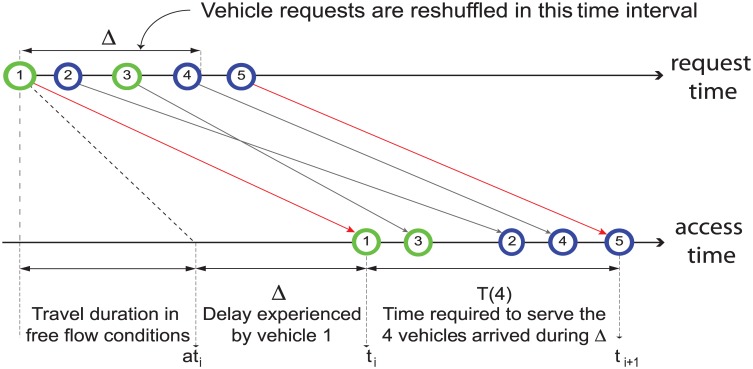
Illustration of the BATCH formation strategy. The value of the time interval used to reshuffle vehicle requests equals the first vehicle delay Δ = (*t*_*i*_ − α*t*_*i*_). Vehicles 2, 3 and 4 are reshuffled. Vehicles in the same flow are represented with circles of same color. Since Vehicle 1 and Vehicle 3 belong to the same flow, Vehicle 3 is given access to the intersection before Vehicle 2. The process is then repeated using Vehicle 5 as reference for Δ. To ensure design goal *ii*), BATCH imposes also an upper bound *N* on the total number of vehicles in a batch. When the number of requests in a batch is 1, BATCH is equivalent to FAIR.

Based on the observation that *T*_1_ < *T*_2_ and aiming at maximizing capacity, the BATCH strategy processes vehicle requests in batches. The goal is forming platoons of vehicles incoming from the same direction that can be served in a short time period *T*_1_—see S1 Supporting Information for details. In the process of jointly handling a batch of intersection access requests, strategy BATCH divides the requests into two groups according to the respective flows, and sequentially gives intersection access to the two groups. The batch formation strategy is reported in Fig 2, and serves a two-fold goal: *i*) since batching requests might increase vehicle delay variance with respect to the FAIR policy, form batches of size larger than 1 only when the system load is increasing, and vehicles start experiencing delays; and *ii*) address the case of two flows with very different loads, with one high-load flow potentially starving the light-load one. The batch formation strategy guarantees also that no vehicle exceeds the prescribed speed limit when approaching the intersection (S1 Supporting Information)—a situation possibly caused by uncontrolled vehicle re-shuffling.

Characterizing the capacity and delays of BATCH requires one to extend existing queuing theory tools [13–22] to the unexplored realm of continuous time and batch-size dependent service times (S1 Supporting Information). Defined *N* as the upper bound on the number of vehicles that can be served in a batch, the obtained capacity value *C*_*B*_(*N*) is an increasing function of *N*, with $\lim_{N\to \infty }C_{B}\left(N\right)=\frac{1}{T_{1}}$. Observing that $\frac{1}{T_{1}}$ corresponds to the capacity of a single road without intersections as dictated only by safety considerations, we can conclude that BATCH converges to optimal capacity.

**Fig 3:**
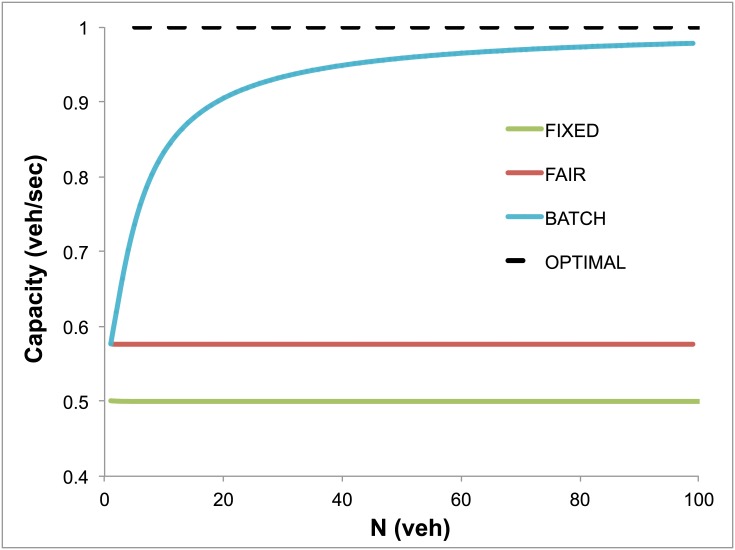
Slot-based intersection control doubles capacity. The capacity of three control strategies is compared: FAIR, the slot-based strategy with first-come-first-serve service policy; BATCH, the slot-based strategy with adaptive vehicle platooning; and FIXED, the traffic light strategy with fixed cycle duration. The service rate of BATCH is twice that of FIXED, and converges to the optimal capacity as the value *N* of the maximal number of platooned vehicles increases. Optimal capacity is estimated assuming a single road without intersections, as dictated only by safety considerations. The service rate of FAIR is only marginally larger than that of FIXED.

A comparison with fixed cycle traffic light (FIXED) systems (Fig 3, Table 1, and S1 Video) shows that service rates of BATCH are superior, asymptotically doubling service capacity with respect to FIXED as *N* increases. This is a very notable result, since it is well known that even small improvements in capacity have multiple times an effect on travel times [24]. The capacity of the FAIR strategy is only marginally higher than the one achieved by FIXED (see Fig 3). The delay achieved by the FAIR and BATCH strategies is dramatically lower than that provided by FIXED.

**Table 1:** Average delays vary dramatically with the control strategy. Expectation and variance of delay for the different strategies and for varying vehicle arrival rates: FIXED, FAIR, and BATCH. FIXED statistics are computed according to [23]. Slot based control strategies dramatically reduce average delay with respect to traffic light control. Delay variance is reduced to a greater extent, indicating that slot based control strategies can lead to more predictable travel times.

Rate (*veh*/*s*)	Average delay (*sec*)			Delay variance (*sec*^2^)		
	FIXED	FAIR	BATCH	FIXED	FAIR	BATCH
0.3	5.45	1.05	0.95	26.21	2.61	1.20
0.4	10.13	2.12	1.63	92.89	7.36	2.15
0.49	99.76	5.06	2.57	7504.41	28.66	3.34

## Discussion

This article extends queuing theory to the realm of continuous time and batch-size dependent service times, with applications in as diverse fields as computing, telecommunication, and facility design.

The theoretical framework presented in this article allows the characterization of intersection performance (S1 Supporting Information) as a function of system features. Geometric parameters have an important effect: for instance, the reduction of vehicle length and intersection width improves capacity and delay. More importantly, the development of autonomous transportation might in itself bring additional benefits when vehicle response times are reduced (S1 Supporting Information).

Results of our analysis highlight that transitioning from traffic lights to SIs could result in a up to 2-fold increase in capacity, and even more dramatic reductions in delay expectation and variance. Such impressive performance improvements can be intuitively understood, as in SIs the “set up phase” is much shorter than in the case of traffic lights. In the analyzed scenario, the “set up phase” corresponds to the difference between *T*_2_ and *T*_1_, i.e., it is about 1.47 *sec* (S1 Supporting Information): a factor of 5 shorter than the typical values used for traffic lights. Since the setup time is so short, the frequency of switching between “phases” does not affect performance as in the case of traffic lights. Thus, the switching frequency can be about a factor 5 higher than in a TL system, dissolving the very notion of “phase” typical for traffic lights into that of single-vehicle slots. Furthermore, vehicles approaching an SI are not grouped in queues near the intersection, but uniformly spread along the road thanks to speed control. As such, they do not need to slow down through a “set up phase”—a fact that further contributes to improved performance. In short, the higher performance of SIs when compared with traffic lights comes from their increased flexibility, finer granularity in merging traffic flows, and better usage of road space.

These features could have a major positive effect at the network level. It is well-known that the highly nonlinear dynamics typical of road networks are triggered when congestion occurs in one or more network bottlenecks [5]. The doubling of bottleneck capacity, as promised by SIs, has the potential of significantly reducing overall congestion and improving the stability and predictability of traffic. In terms of predictability, it is important to observe that SIs dramatically reduce not only average delay versus traffic lights, but also delay variance (Table 1). Delay values are highly concentrated around the average, further enhancing travel time predictability. Similarly, autonomous driving can substantially reduce the non-linear flow dynamics as a function of vehicle density, which is known to be caused by different human driving styles [25, 26].

Further work would be needed to scale up our analysis to a network of road intersections. Unlike in the case of traffic lights, such scaling is feasible from a computational point of view, since all SI algorithms presented herein require minimal computational effort. SIs would probably also have beneficial effects on car emissions, as they would reduce the “stop-and-go” effect induced by traffic light queuing.

It is interesting to observe that the optimal BATCH strategy defined in this paper leverages the slower-is-faster effect. The slower-is-faster effect, which has been observed in fields as diverse as traffic [24], pedestrian movement [27], production and logistics [28], etc., arises when the apparently detrimental choice of a slower initial speed eventually results in a faster service time. BATCH exploits this effect since, by “re-shuffling” vehicles in a batch, it slows down some of the vehicles (those belonging to the yielding flow) but increases throughput, leading to an overall reduction of the average delay. This article formally demonstrates that the slower-is-faster control principle also applies to slot-based intersections.

## Materials and Methods

The SIs intersection management algorithms are based on the following operations. As they enter the system, vehicles issue intersection access requests to an entity called Intersection Manager using wireless communication. A request from vehicle *V* is accompanied by the earliest possible arrival time *at*_*V*_ of vehicle *V* at the intersection (computed according to speed limit and safety constraints). FAIR processes vehicle requests individually, based on a FCFS policy. When processing request from vehicle *V*, FAIR assigns to *V* an intersection access time *t*_*V*_ ≥ *at*_*V*_, where *t*_*V*_ is computed accounting for safety considerations based on either tailgate or vehicle stopping distance depending on whether the vehicle *V*′ accessing the intersection immediately before *V* belongs to the same or to the other flow.

BATCH splits time into a series of consecutive time intervals of variable duration, and collectively processes all requests in each such interval. The duration of the time interval used to shuffle vehicle requests is set to be equal to the delay experienced by a given vehicle, with an upper bound *N* on the total number of requests—see Fig 2. Let $V^{N}=\left\{V_{1}^{N},\ldots ,V_{k}^{N}\right\}$ and $V^{E}=\left\{V_{1}^{E},\ldots ,V_{h}^{E}\right\}$ be the ordered lists of requests arrived in a batch from vehicles in the *N* and *E* flow, respectively, and assume without loss of generality that $V_{1}^{N}$ arrived before $V_{1}^{E}$. BATCH assigns ordered intersection access times to each vehicle from $V^{N}$, accounting for safety considerations, and then assigns ordered intersection access times to each vehicle from $V^{E}$, again accounting for safety considerations.

In both FAIR and BATCH, assigned intersection access times are then communicated to approaching vehicles using wireless communication. Generalization of FAIR and BATCH to the case of 12 trajectories, 4-roads, 2-lanes scenario is described in S1 Supporting Information.

For the sake of comparison, in the analysis we also consider a Fixed Cycle Traffic Light system, whose delay performance has been formally derived in [23]. The cycle of the traffic light is composed of two phases of *fixed* (but possibly different) duration: in the first phase, green light is given to the *N* flow, and red light is given to the *E* flow; in the second phase, the opposite holds. For simplicity, we assume that for both flows the amber light phase is included in the respective green light phase.

## Supporting Information

S1 Supporting Information Revisiting street intersections using slot based systems—*supplementary information*. (PDF)

S1 Video Side by side comparison of slot based intersection and traffic light system. (MOV)
