# Supplementary material for: Revisiting Street Intersections Using Slot-Based Systems
Source: PLoS One. 2016 Mar 16;11(3):e0149607. doi: 10.1371/journal.pone.0149607 (PMC4794159; doi:10.1371/journal.pone.0149607)
Supplement: S1 Supporting Information — (PDF) [file pone.0149607.s001.pdf]

Revisiting street intersections using slot based systems
Supplementary Information

September 11, 2015

1 Material and methods

1.1 Safety considerations

The first level of safety considered in our framework is that of a single vehicle moving along a pre-defined trajectory. Here, safety considerations dictate the maximum possible speed of a vehicle along the trajectory, where the maximum speed depends not only on speed limit regulations, but also on the radius of curvature of the trajectory. While the presented analytical framework is independent of any speed setting for vehicle trajectories, the specific speed values used in the study are reported in the next section. With first-level safety implemented, we can regard vehicles as entities traveling along a pre-defined trajectory without deviations.

The second safety level is that of two vehicles traveling along their trajectories. Second-level safety requirements are designed to ensure safety of a vehicle V traveling along trajectory T_V also in the situation in which vehicle V' traveling along trajectory $T_{V'}$ comes to a complete stop. Here, we first observe that in our framework vehicles are assumed to travel along a number of fixed, pre-defined trajectories. An *incompatibility relationship* is defined between pairs of trajectories depending on whether they cross each other in the intersection area. Non-crossing trajectories are designed in such a way that vehicles traveling along these trajectories never hit each other and maintain adequate lateral inter-vehicle distance, even if they simultaneously access the intersection. For this reason, any two trajectories that are not bound by the incompatibility relationship are called *compatible*.

Second-level safety requirements can be expressed based on the notion of *vehicle stopping distance* d_{stop}, which can be informally defined as the distance between the point at which the driver (or, the vehicle control system in case of self-driving vehicles) detects a dangerous situation, and the point at which the vehicle comes to a complete stop – see next section for formal definition. Consider a vehicle V' traveling along trajectory $T_{V'}$, and coming to a complete stop somewhere within the intersection area. Consider now a vehicle V approaching the intersection along trajectory T_V, and assume $\{T_{V'}, T_V\}$ are incompatible trajectories. From the viewpoint of vehicle V, V' is seen as an obstacle obstructing the intersection area. Hence, in order to ensure second-level safety we mandate that vehicle V must be at distance at least d_{stop} from the intersection area during the time needed by V' to clear the intersection area.

A special case is that of vehicles $\{V, V'\}$ traveling along the *same* trajectory. In this case, we are in a typical car following situation, and second-level safety can be expressed accounting for the fact that the relative speed between vehicles $\{V, V'\}$ is in general much smaller than the traveling speed. Safety is then defined in terms of the *tailgate distance* d_{tail}, which is designed to avoid tailgating and has the property of ensuring a time-separation between vehicles which is independent of their speed, and for this reason it is known as the *two seconds rule* in the traffic engineering community – see next section for formal definition. An important consideration about the two second-level safety criteria is that safety based on distance d_{stop} significantly penalizes the volume of traffic that can be managed by the intersection with respect to safety based on distance d_{tail}, since $d_{stop} > d_{tail}$ (see next section).

Finally, we observe that third-level safety requirements might be imposed to consider the situation in which a vehicle deviates from its prescribed trajectory due to an unexpected event (e.g., exploding tyre). A straightforward way of ensuring third-level safety is granting access to the intersection area to a single vehicle at a time, and to regulate inter-vehicle distances according to distance d_{stop}. Alternatively, one might impose a relatively larger distance separation between trajectories, possibly reducing the number of mutually

compatible trajectories. Analyzing the impact of third level safety requirements on intersection management performance is outside the scope of this paper.

1.2 Estimating the effect of geometric parameters on SIs algorithm performance

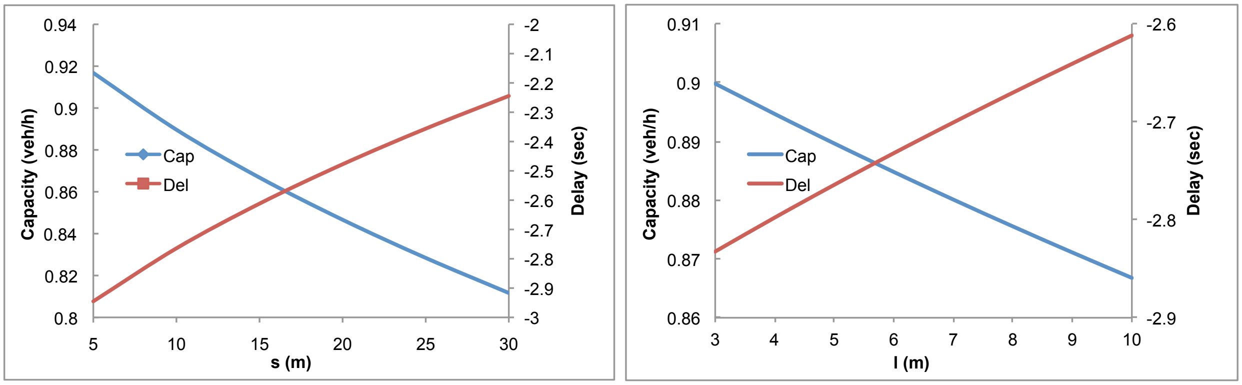

Figure S1: Effect of intersection width s and vehicle length ℓ on BATCH performance: capacity (primary axis, left) and average delay (secondary axis, right).

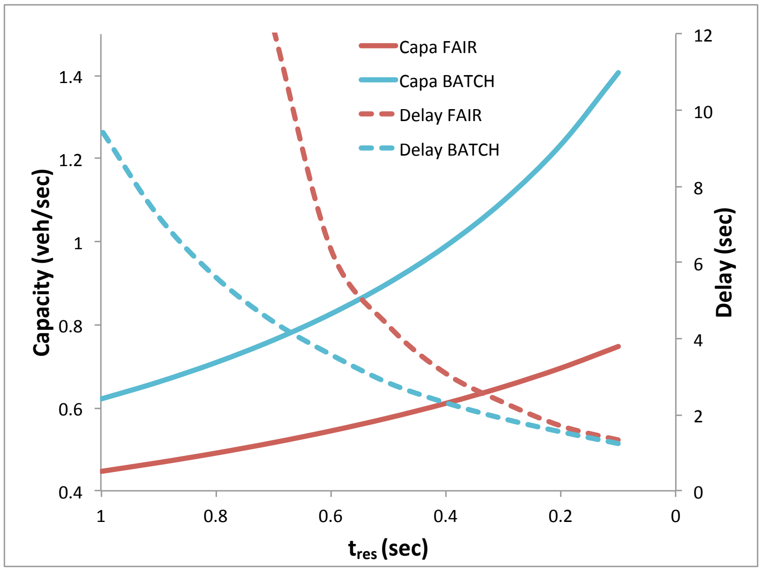

Figure S2: Effect of advancement in self-driving technology on FAIR and BATCH performance: capacity (primary axis, left) and average delay (secondary axis, right) with decreasing values of the response time t_{res}.

In Figure S1, we report the capacity and expected delay provided by the BATCH algorithm for increasing values of s (intersection width) and ℓ (vehicle length). As seen from the figure, increasing intersection width and car length have negative effects on both capacity and average delay.

Figure S2 reports the effect of self-driving technology advancement on capacity and delay: reducing the response time from 1 *sec* (typical human driver) to .5 *sec* (conservative estimation of response time with automated driving) results in a 1.5-fold capacity increase and in a 2-fold delay reduction.

1.3 Derivation of inter-vehicle intersection access times

In this Section, we present a methodology for deriving the minimal inter-vehicle intersection access times T_1 and T_2 used in the theoretical framework. We recall that T_1 is the inter-vehicle intersection access time when

two subsequent vehicles belong to the same flow, while T_2 refers to the situation in which the two vehicles belong to different flows.

Time T_1 can be readily derived from the expression for tailgate distance d_{tail}, and it is independent of the speed v of the approaching vehicles:

$$T_1 = \frac{d_{tail}}{v} = \frac{v(t_{res} + \Delta)}{v} = t_{res} + \Delta \ .$$

The results reported in the main text are obtained by setting $T_1 = t_{res} + \Delta = 1$ *sec*, which reasonably upper bound the response time of an autonomous driving system including the tolerance parameter Δ.

Differently from T_1, time T_2 depends on the vehicle speed v, and it computed assuming that the second vehicle approaching the intersection is at distance d_{stop} when the tail of the first vehicle leaves the intersection area. Thus, the inter-vehicle intersection access time can be written as:

$$T_2 = T_2(v) = \frac{d_{stop}(v)}{v} + t_{xing}(v) = t_{res} + \frac{v}{2 \cdot a_{brake}} + \frac{\ell + s}{v} \ ,$$

where $t_{xing} = t_{xing}(v) = \frac{s}{v}$ is the time required by the first vehicle to exit the intersection area.

Time $T_2(v)$ is a continuous function in v, whose minimum is achieved at

$$v^* = \sqrt{2 \cdot a_{brake}(s + \ell)} \ .$$

Hence, in the analysis T_2 is computed as $T_2 = T_2(v^*)$. More specifically, setting $s = 10\ m$, $\ell = 5\ m$, and $a_{brake} = 7.72\ m/sec^2$, we obtain $v^* = 15.2184\ m/sec$, which results in $T_2 = 2.4713$ *sec* when t_{res} is set to 0.5 *sec*.

Notice that, for practical values of the system parameters such as the ones used above, we have $d_{tail} < d_{stop}(v)$ for any $v > 0$, and, in particular, $d_{tail} < d_{stop}(v^*)$.

1.4 Traffic light modeling

1.4.1 Fixed cycle traffic light analysis

The analysis of the delay (also called waiting time in the following) in the fixed cycle traffic light (FIXED) system can be found in (29). The approach of (29) allows computing the probability generating function of the stationary waiting time distribution, from which first and second order moments can be computed. In the model considered, time is discretized into slots of equal duration, and vehicles are assumed to arrive at the end of each slot. The duration g of the green phase is expressed as an integer number of slots, where a slot is defined as the amount of time needed by a delayed (queued) vehicle to depart from the queue.

The analysis in (29) can be used to estimate the average and variance of the delay in the FIXED system, which is expressed in units of slot. Thus, in order to compare the delay in IM and FIXED systems, we need to define the slot duration in seconds. To this purpose, we use the analysis in Section 1.4.3. For instance, if $g = 5$ as in the numerical evaluations reported in (29), we have a green phase duration of 10 *sec* according to the analysis of 1.4.3, implying that the length of a slot is 2 *sec*.

Notice that the analysis of (29) *underestimates* the actual delay for the following reasons:

– time is discretized; hence, all vehicles are assumed to arrive at the end of the slot, which implies a slight underestimation of delay;

– in the model, vehicles arriving at the intersection and finding an empty queue are assumed to experience 0 delay;

– in the model, slots have fixed duration, while the analysis reported in Section 1.4.3 shows that in a real system slots have different durations, with relatively longer slots occurring at the beginning of the queue. Thus, if one considers the *average* duration of a slot to define the slot duration as in (29), the actual delay is underestimated (the probability of finding an empty queue being higher in that case).

1.4.2 Numerical comparison of the slot-based algorithms and FIXED

In (29), we can find the average and variance of the delay for the FIXED system with a five slot green and red phase. We recall that we set the slot duration to $\delta = 2sec$. As far as the arrival rate is concerned, we use the values found in (29). Since we here consider a single lane traffic light, the incoming rate to our intersection must be twice the one to the traffic light. Also, because all the quantities are measured in terms of slot duration, the average arrival rate in (29) must be divided by δ, while $\mathbb{E}[W]$ and $\mathrm{Var}[W]$ must be multiplied by δ and δ^2 respectively. Taking all the above into account, we get the results reported in Table 1 of the main text for mean and variance of the delays.

1.4.3 Computing the slot duration in the FIXED system

We assume here that whenever the N flow is in the green light phase, the E flow is in the red light phase, and vice-versa, and that the flows have equal priorities, so that their green light phases have exactly the same durations.

Furthermore, we let d_{sep} be such that the head of the first vehicle in the queue is at a distance of $d_{sep}/2$ from the intersection area, and such that, for every other vehicle V in the queue, the head of V is at a distance of d_{sep} from the tail of the V's predecessor in the queue. Moreover, for each vehicle V and each time instant t, we let $FP_V(t)$ denote the footprint of V at t, which is defined as the stretch of road of length $\ell + d_{sep}$ centered at the vehicle's (geometric) center. Thus, the head and tail of a vehicle's footprint are the points that lie $(\ell + d_{sep})/2$ units ahead and behind the vehicle's center, respectively.

Regarding the vehicle dynamics during the green phase, we assume that at the time instant a road's light turns green, the first vehicle in the queue starts to accelerate. We are assuming no control system's response time here, since we are considering a scenario in which the vehicle's control system is notified of the transition to the green phase ahead of time. Consistently with the notion of tailgate distance, the i-th vehicle in the queue, with $i = 2, \ldots M$, starts accelerating T_1 seconds after the $(i-1)$-th vehicle. Each vehicle advances with constant forward acceleration a_f until it reaches cruise speed $v = v_{max}$ as dictated by the speed limit, and then maintains constants speed v throughout. Finally, we consider vehicle V as having left the queue once the tail of its footprint has entered the intersection area; also, we consider V as having left the intersection once the tail of its footprint has exited the intersection area.

We are now ready to compute the time required for M vehicles to clear the intersection. To this purpose, we use $V_1, \ldots, V_M$ to denote the vehicles that will leave the queue during the green phase, in the order in which they leave the queue (recall that each incoming road is a FIFO queue), i.e., V_1 denotes the first vehicle to leave, and V_M is the last vehicle to leave. Furthermore, note that the time and the distance required to accelerate from rest to cruise speed, denoted t_{accel} and d_{accel}, respectively, are given by

$$t_{accel}(v) = \frac{v}{a_f} \quad \text{and} \quad d_{accel}(v) = \frac{v^2}{2\,a_f} \ .$$

Let us consider the i-th vehicle in the queue, whose tail is at distance

$$d_{exit}(i) = (i-1)(\ell + d_{sep}) + \frac{d_{sep}}{2} + s + \ell$$

from P_{out}.

Hence, if $d_{exit}(i) \leq d_{accel}(v)$, then V_i is still accelerating at the moment it exits the intersection. On the other hand, if $d_{exit}(i) > d_{accel}(v)$, then V_i reaches cruise speed before exiting the queue. To make this distinction more explicit, let the $\hat{\imath}$ denote the position in the queue at which transition occurs, which can be obtained by solving equation $d_{exit}(i) = d_{accel}(v)$ for variable i. After straightforward algebraic manipulations, we obtain:

$$\hat{\imath}(v) = \frac{\frac{v^2}{2\,a_f} - s + \frac{d_{sep}}{2}}{\ell + d_{sep}} \ . \tag{1}$$

Let $\theta_M(v)$ denote the duration of the interval between the moment vehicle V_M starts accelerating and the moment it exits the intersection. Since vehicles in the queue start every T_1 seconds, the amount of time elapsed from the time instant the light turns green to the moment V_M exits the intersection, denoted by

$t(M, v)$, equals $T_1(M-1) + \theta_M(v)$. Now, consider the case $M \le \lfloor \hat{i}(v) \rfloor$. Then, from basic kinematics, we see that $\theta_M(v)$ obeys

$$\frac{a_f(\theta_M(v))^2}{2} = d_{exit}(M)$$

and so it follows that

$$t(M, v) = T_1(M-1) + \sqrt{\frac{2\, d_{exit}(M)}{a_f}}, \quad \forall\, M \le \lfloor \hat{i}(v) \rfloor \tag{2}$$

Next, consider the case $M > \lfloor \hat{i}(v) \rfloor$. Then,

$$\theta_M(v) = t_{accel}(v) + \frac{d_{exit}(M) - d_{accel}(v)}{v}\ , \quad \forall\, M > \lfloor \hat{i}(v) \rfloor \tag{3}$$

since in this case $\theta_M(v)$ exceeds $t_{accel}(v)$ by the amount of time it takes V_M to cover distance $d_{exit}(M) - d_{accel}(v)$ while traveling at speed v.

Notice that, thanks to self-driving technology, the green phase for the other direction can start soon after vehicle V_M exits the intersection, implying that the duration of one complete cycle of each queue (i.e., a green-amber-red-amber cycle), denoted $t_{cycle}(M, v)$, is given by

$$t_{cycle}(M, v) = 2\, t(M, v) \quad \forall\, M \ge 1, \quad \forall\, v > 0\ .$$

The *average vehicle service time* associated with each queue, denoted $t_{srv}(M, v)$, is then given by the ratio $t_{cycle}(M, v)/M$, and equals the slot duration used in the analysis of (29).

1.5 Intersection management algorithms

In this paper, we consider two slot-based algorithms: FAIR and BATCH. Since FAIR can be derived from BATCH by setting the upper bound N on the maximum number of vehicles in a batch to $N = 1$, in the following we describe only algorithm BATCH. The general setting is the following:

- a list of earliest time arrivals modeling vehicle arrivals is generated, following a Poisson process of given intensity λ; the arrival times are assumed to be smaller than a given time T;
- those arrivals are assigned to a flow, with probability d for flow N and $(1-d)$ for flow E.

The requests can be represented as $(\{V, at_V, X_V\})_{V \in C}$, where C is the set of vehicles, $0 \le at_V \le T$ the earliest arrival time of vehicle V at the intersection and $X_V \in \{N, E\}$ the flow vehicle V belongs to. The list of vehicle arrivals is assumed to be sorted. A solution to the intersection management problem is a list $(\{V, t_V\})_{V \in C}$ of access times to the intersection, for each vehicle. Those times must verify the safety constraints as described in the main text, including the fact that vehicle passing is not allowed, i.e., if $at_V < at_{V'}$ and $X_V = X_{V'}$, then $t_V < t_{V'}$. For instance, if V and V' belong to the same flow, we must have $|t_V - t_{V'}| \ge T_1$. Their respective access times are communicated to the vehicles on the road. They then adjust their speed to reach the intersection at the right time and at the right speed (32), for optimality that speed is to be equal to v^* defined in Section 1.3.

1.5.1 BATCH for two-lanes scenario

As mentioned in the main text, an important issue to take into account when reshuffling that certain vehicles might be granted access to the intersection before their earliest arrival time, which implies vehicle speeds exceeding the speed limit. In order to avoid this, the time interval used to re-shuffle vehicle requests is adjusted to the traffic load as follows. Initially, vehicle requests are dealt with on a FCFS basis. As soon as a vehicle V experiences a delay Δ (or, in other words, is granted access to the intersection at a time t_V strictly larger than its earliest arrival at_V), the time interval is set to $\Delta = (t_V - at_V)$, and all the vehicles arriving during that interval are processed as a single batch in BATCH. At the end of that batch, the vehicles are once again dealt with on a FCFS basis, until another vehicle is delayed and the process is reiterated. As mentioned in the main text, in order to avoid giving access to the intersection to potentially boundless lanes of vehicles from the same direction, we limit the maximum number of reshuffled cars to N. If N is reached during a single batch, the $(N+1)$-th car (which is necessarily delayed) will serve as a pivot for the next reshuffling. It is possible to prove the following simple proposition

Proposition 1. *In BATCH, no vehicle exceeds the speed limit.*

Proof. It is clear that as long as a vehicle reaches the intersection after its earliest arrival time, it will respect the speed limit. Let $\overline{V}$ denote the first delayed vehicle. Its access time is $t_{\overline{V}}$ and $\Delta = t_{\overline{V}} - at_{\overline{V}}$, and the considered batch is $B = \{V \in C, at_{\overline{V}} < at_V \leq t_{\overline{V}}\}$. A vehicle V from B is granted access to the intersection after vehicle $\overline{V}$, hence $t_V > t_{\overline{V}} \geq at_V$, which concludes the proof. □

The pseudocode for BATCH is reported below. Let us point out the fact that a batch is created for every vehicle. In the case where there is no delay ($t_l = at_{\overline{V}}$ in the code), it only contains vehicle $\overline{V}$, so that the serving policy becomes equivalent to FCFS.

BATCH ALGORITHM

let V_0 be the first vehicle
set access time and direction of the last vehicle: $t_l = at_{V_0}$, $d_l = X_{V_0}$
set number of processed vehicles to 1: $n_p = 1$
while $n_p < |C|$
  let $\overline{V}$ be the $(n_p + 1)$-th vehicle in C
  set $T_{sep} = T_1$ if $X_{\overline{V}} = d_l$, T_2 otherwise
  set $t_l = max(t_l + t_{sep}, at_{\overline{V}})$
  create batch $B = \{V \in C, at_{\overline{V}} \leq at_V \leq t_l = at_{\overline{V}} + \Delta\}$, with $|B| \leq N$
  process batch B using *BatchProcessing* with $t_f = t_l$
  let V_l be the last vehicle processed in B
  set $t_l = t_{V_l}$, $d_l = X_{V_l}$, $n_p = n_p + |B|$

The pseudocode of the *BatchProcessing* algorithm is reported below.

BATCHPROCESSING ALGORITHM

set access time for the next vehicle to $t_f = t_l$
let X be the flow of the first vehicle in B
for all vehicles V in batch B with flow X
    set $t_V = t_f$
    if V is the last vehicle from flow X:
        if there is a vehicle from flow $Y \neq X$
            $t_f = t_f + T_2$
    else
        $t_f = t_f + T_1$
for all vehicles V in batch B with flow $Y \neq X$
    set $t_V = t_f$
    if V is *not* the last vehicle from flow Y:
        $t_f = t_f + T_1$

1.5.2 BATCH for a general intersection

We now consider a general type of intersection, described by its *compatibility network*. The compatibility network is a network $\mathcal{CN} = (\mathcal{T}, E)$ where $\mathcal{T}$ is the set of all possible flows approaching the intersection, and $(T_X, T_Y) \in E$ if and only if the trajectories followed by vehicles belonging to trajectories T_X and T_Y do not cross each other at the intersection, and are thus compatible. Figure S3-A reports a portion of the compatibility network of a four roads, eight lanes intersection with twelve possible incoming flows: for each of the four directions $\{N, E, S, W\}$, we have three possible flows corresponding to vehicles following a straight (S), left (L), or right (T) trajectory at the intersection.

A set of trajectories $\mathcal{T}$ and of lanes $\mathcal{L}$ is defined. For each $X \in \mathcal{T}$, $\mathcal{T}_X$ denotes the set of directions compatible with X as defined above, and L_X the lane corresponding to trajectory X. The list of requests is still represented as $(\{V, at_V, X_V\})_{V \in C}$. This time, however, X_V lives in $\mathcal{T}$. The algorithm requires two steps: defining a FCFS policy for compatibility networks, and write the reshuffling of vehicles within the batches.

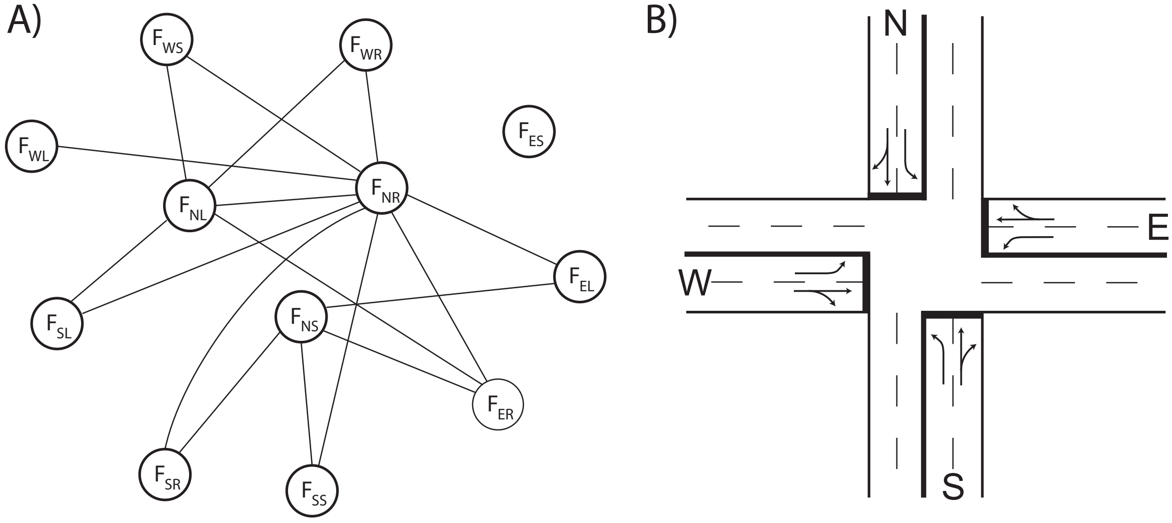

Figure S3: 12 trajectories, 8 lanes intersection. (A) Compatibility network for trajectories coming from the North direction. (B) Intersection configuration

Let us consider a set of requests, and process it on a FCFS basis. For a given vehicle V, the earliest allowed arrival t_a is the maximum between its own earliest arrival time at_V, and the access time of the vehicle preceding it in its lane (those times are saved in a list denoted A in the pseudocode), increased by T_1. Then, let P_i denote the set of vehicles already processed and with a trajectory which is incompatible with that of V. The problem then simply reduces to finding a time $t \geq t_a$ that is separated from all access times in P_i by at least T_2. Formally, this algorithm can be written as follows.

FAIR ALGORITHM

0. create a 'last access time per lane' list $A = [-T_1, \cdots, -T_1]$ [a]
1. initialize the list of processed vehicles $P = \varnothing$
2. for vehicle $\overline{V}$ in C (sorted by increasing at_V)
3. let $L = L_{X_{\overline{V}}}$ be the lane $\overline{V}$ belongs to
4. set $t_a = max(at_{\overline{V}}, A[L] + T_1)$
5. let $P_i = \{V \in P, X_V \notin \mathcal{T}_{\overline{X}}\}$ be the vehicles incompatible with $\overline{V}$
6. set $T_i = \{t_V \geq t_a, V \in P_i\}$
7. $t_{\overline{V}} = min\{t \geq t_a : \forall u \in T_i |t - u| \geq T_2\}$
8. add $\overline{V}$ to P
9. set $A[L] = t_{\overline{V}}$

---

[a]Length(A) is the number of lanes

Now that FAIR has been defined, we shall use it in our more general framework. The request list is processed on a FCFS basis. Exactly as in the two-lanes configuration, as soon as a delayed vehicle appears, the vehicles arriving during that time interval are grouped into a batch B. B is then re-ordered: trains of vehicles belonging to the same lane are formed, respecting the order in which lanes appeared in the original batch. For instance, if $B = \{X, Y, X, Z, Y, X, Z\}$, it would become after reshuffling: $B = \{X, X, X, Y, Y, Z, Z\}$. The vehicles belonging to B are processed using the FAIR algorithm described above. The next vehicle is then dealt with on a FCFS basis. If delayed, it also generates a new batch and reshuffling. The pseudocode boils down to the following:

BATCH ALGORITHM

create a 'last access time per lane' list $A = [-T_1, \cdots, -T_1]$
initialize the list of processed vehicles $P = \varnothing$
while $P \subsetneq C$
    let $\overline{V}$ be the earliest vehicle in $C \setminus P$
    compute $t_{\overline{V}}$ using the FCFS algorithm
    create request batch $B = \{\{V, at_V, X_V\}, at_{\overline{V}} \leq at_V \leq t_{\overline{V}}\}$, with $|B| \leq N$
    sort B with respect to L_{X_V}, in order of appearance
    process all the vehicles in B using FCFS with A and P
    set $P = P \cup B$

1.6 Simulation methods and results

1.6.1 Computing capacity for BATCH

Capacity is measured as the maximum arrival rate such that cars requesting access in a given time interval $[\delta_1, \delta_2]$ are processed by the intersection during at most $\delta_2 - \delta_1$ seconds. In practice, this boils down to

1. generate a random list of requests $\{at_1, at_2, \cdots, at_n\}$ with arrival rate λ
2. compute the access times for those vehicles $\{t_1, t_2, \cdots, t_n\}$
3. select a time window $[\delta_1, \delta_2]$
4. find the cars arriving during that time window: $at_i = \min\{at_k \geq \delta_1\}$ and $at_j = \max\{at_k \leq \delta_2\}$
5. find the maximum λ such that $t_j - t_i \leq \delta_1 - \delta_2$

As long as a sufficient number of simulations were used, around 1000, the capacity measured by this method was stable with respect to δ_1 (larger than 100 seconds, to ensure that the stationary regime was reached) and δ_2. The capacity for a given set of parameters is shown in the main text (Figure 3B).

1.6.2 Real life intersection

To compare the performance of FAIR and BATCH with that of an adaptive traffic light system in the case of a non-empty compatibility network, we have simulated the management of a single intersection with four incoming roads from the N, E, S, and W direction, see Figure S3-B. Each road has two lanes per direction, where the left lane in each direction is reserved for vehicles with a "turn left" direction. The intersection width is set to $s = 10$ m, and vehicle length ℓ to 5 m. The length of the roads leading to the intersection is set to 300 m in all directions.

The simulation methodology is as follows. Vehicle inter-arrival times are exponentially distributed, with an average arrival rate of λ vehicles per hour, where λ is a tunable parameter. When a new vehicle arrives, it is assigned an incoming direction and trajectory according to the trajectory mix computed from a sample of real vehicular traffic in the city of Singapore (see below). Once entering the road, the vehicle requests access to the intersection and adjust its speed (32) in order to reach the intersection at the access time computed by the IM system. In case of TL system, the vehicle is governed by a car-following model (33), in free flow it drives at cruising speed $v_{free} = 70$ Km/h till it approaches the intersection; upon approaching the intersection, the vehicle maintains the cruising speed in case of green light and no slow-moving vehicle ahead; otherwise, it reduces its speed in order to come to a complete stop (in case of red/amber light), or to match the speed of the vehicles ahead.

The TL system used in the simulation has a cycle composed of four phases: two phases for "straight" and"right" trajectories, and two phases for "left" trajectories. More specifically the four phases are as follows: *Phase 1*: North Straight, North Right, South Straight, South Right; *Phase 2*: North Left, South Left; *Phase 3*: West Straight, West Right, East Straight, East Right; *Phase 4*: West Left, East Left.

The relative duration of the various phases is determined based on the trajectory mix, as follows. Let p_{XY} be the probability of observing trajectory XY, and let p_{PhaseX} be the sum of the probabilities of all

Origin	North			East			South			West		
Destination	W	S	E	N	W	S	E	N	W	S	E	N
Probability (%)	2.2	11.5	9.5	1.8	4.4	20.5	2.0	2.4	1.5	12.9	25.2	6.1

Table S1: Trajectory mix sampled from the intersection between Commonwealth Av. and North Buona Vista Rd in Singapore.

trajectories in Phase X, i.e.,

$$p_{PhaseX} = \sum_{XY \in PhaseX} p_{XY} \ .$$

The duration of the phases in the cycle is set to be proportional to the probabilities p_{PhaseX} (with a complete cycle duration of 100 seconds). Additionally, to mimic the recent advancements in TL technology, the green phase is automatically reduced if no more vehicles are awaiting at the intersection for the "green light" flows (it thus becomes a very basic adaptive traffic light in light of (36). We do not consider here car-to-car communication (34) nor speed optimization when approaching a red light (35), as those are - at least partly - encompassed in our model.

A single simulation experiment consists in generating arriving vehicles with a fixed rate λ for an interval of 20 min, which is found to be sufficient to reach stationary conditions. The trajectory mix used in the simulations is based on the traffic observed in a real intersection in the city of Singapore.

Estimating the trajectory mix at a real intersection. We have considered the intersection between Commonwealth Av. and North Buona Vista Rd, which is known to be a heavily loaded intersection. It also has the nice property of being quite far from its adjacent intersections, hence appeared as a good test subject for our algorithms. In order to estimate the trajectory mix, we have considered the dataset composed of approx. 400,000 trips performed by around 13,000 taxis in Singapore during a day of February 2011. It contains the GPS location of each taxi sampled with a varying frequency, with typically one sample every $20 - 30$ sec. The dataset was pre-processed to consider only trips going through the intersection of interest, which amounted to about 2,000 trips. Then, for each relevant trip, a trace of the trajectory undertaken by the taxi at the intersection was kept. The recorded number of taxi trips for each of the possible 12 trajectories was then used to compute the trajectory mix, which is reported in Table S1.

1.6.3 Simulation results

The performance in terms of delays average and variance, as well as service rate are reported in Figure S5 in Extended Data. IM algorithms give impressive improvements vs. traffic light systems: the capacity is doubled for BATCH compared to an adaptive Traffic Light (aTL), where the green phase duration equals the time required for all the vehicles waiting to cross the intersection. When the vehicle arrival rate gets close to the traffic light capacity, the average delay experienced by vehicles in both IM algorithms remains smaller than 6 seconds. It is also worth noting on Figure S5 in Extended Data that at equal load, FAIR provides smaller delay standard errors than aTL and, at a smaller scale, than BATCH, which confirms the intuition that it is the 'fairer' system.

1.7 Theoretical results

In this section we prove the theoretical results from the main text. Unless otherwise stated, the reference configuration is the two-lanes scenario, as described in Figure 1 of the main text.

1.7.1 FAIR

As described in the main text, FAIR can be described as an M/G/1 system in queuing theory (5). This allows us to readily derive the capacity, as well as the first and second order moments of the delay (or *waiting time* in the queuing literature). The intersection capacity achieved by the FAIR strategy equals the inverse of the average service time (5), i.e.,

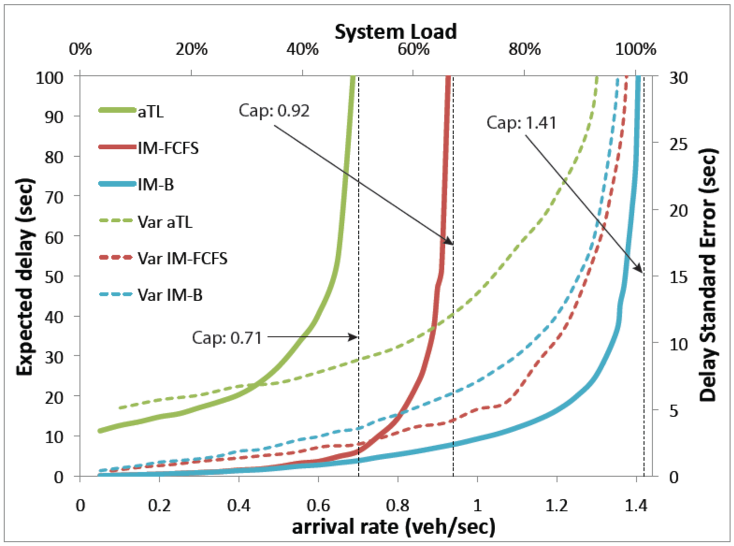

Figure S4: Performance of the various intersection management strategies in the 4-roads, 2-lanes scenario. Primary axis: Capacity and delays for aTL(adaptive Traffic Light) and the algorithms FAIR and BATCH, for the real trajectory mix measured in Singapore. Secondary axis, dashed lines: Delay standard error as a function of the load for the same three intersection management strategies.

$$C_{FCFS} = \frac{1}{\mathbb{E}[T]} = \frac{1}{p \cdot T_1 + (1-p) \cdot T_2} \ ,$$

while the first and second order moments of the delay are

$$\mathbb{E}[D_{FCFS}] = \frac{\lambda \mathbb{E}[T^2]}{2(1-\rho)}$$

and

$$\mathbb{E}[D_{FCFS}^2] = 2\mathbb{E}[D_{FCFS}]^2 + \frac{\lambda \mathbb{E}[T^3]}{3(1-\rho)} \ ,$$

where $\rho = \frac{\lambda}{C_{FCFS}}$ is the *load* of the system.

1.7.2 BATCH

Let us describe thoroughly the BATCH process of serving requests from a queuing theory viewpoint. Ignoring the - irrelevant in this section - fact that requests are performed in advance (when cars enter the road in our case), the system boils down to a server that processes customers in batches, with customers arriving indivudually. The service capacity of each batch is constant and equal to N. Batches are served as soon as the server is idle, meaning that if no customer is available at the end of the n-th batch, then the $(n+1)$-th batch only contains one vehicle, the next one to arrive. From the BATCH algorithm point of view, such a car has a zero delay. The service time T of a batch depends on its size n, hence is denoted $T(n)$. The distribution $B_n(t)$ of $T(n)$ depends on the strategy chosen to grant access to the intersection. In technical terms, this system is a bulk-service queue with batch-size-dependent service: M / GrY / 1, a type of queue whose performance has not yet been characterized in the literature. In the following, we close this gap in the queuing theory literature and present the formal proof of the capacity formula derived for the BATCH algorithm in the two-lanes configuration.

Capacity. Let $(D_t)_{t\geq 0}$ be the number of vehicles present in the system at time t, and let $(t_i)_{i\in\mathbb{N}}$ be the time instant of the i-th batch departure from the system. Then $m_i \equiv D_{t_i^+}$ for $i = 1, 2, \ldots$ is an embedded Markov

chain (19, 20, 37) of the process $(D_t)_{t\geq 0}$. Let us also define $(a_i)_{i\in\mathbb{N}}$ as the number of customers arriving during the service of the i-th batch. Since arrival times follow a Poisson process with rate λ, we have

$$\mu_{j,n} \equiv P(a_i = j|\{n \text{ cars in batch } i\}) = \int_0^\infty e^{-\lambda t}\frac{(\lambda t)^j}{j!}dB_n(t)$$

It follows from the way batches are processed that

$$m_{i+1} = \begin{cases} [m_i - N]^+ + a_i & \text{if } m_i > 0 \\ a_0 & \text{if } m_i = 0 \end{cases}$$

where a_0 is a random variable following the distribution given by $\mu_{j,1}$. The stationary transition matrix for m_i is the following

$$Q = \begin{pmatrix} \mu_{0,1} & \mu_{1,1} & \mu_{2,1} & \mu_{3,1} & \cdots & \mu_{i,1} & \cdots \\ \mu_{0,1} & \mu_{1,1} & \mu_{2,1} & \mu_{3,1} & & \mu_{i,1} & \\ \mu_{0,2} & \mu_{1,2} & \mu_{2,2} & \mu_{3,2} & & \mu_{i,2} & \\ \mu_{0,3} & \mu_{1,3} & \mu_{2,3} & \mu_{3,3} & & \mu_{i,3} & \\ \vdots & & & & \ddots & & \\ \mu_{0,N} & \mu_{1,N} & \mu_{2,N} & \mu_{3,N} & & \mu_{i,N} & \\ 0 & \mu_{0,N} & \mu_{1,N} & \mu_{2,N} & & \mu_{i-1,N} & \\ 0 & 0 & \mu_{0,N} & \mu_{1,N} & & \mu_{i-2,N} & \\ 0 & 0 & 0 & \mu_{0,N} & & \mu_{i-3,N} & \\ \vdots & & & & & & \ddots \end{pmatrix}$$

The capacity for this batch-size dependent queue is $\frac{N}{\mathbb{E}[T(N)]} = \frac{N}{\int_0^\infty tdB_N(t)}$. Letting $\rho = \frac{\lambda\mathbb{E}[T(N)]}{N}$, we can prove the following lemma:

Lemma 1. *The chain $(m_i)_{i\in\mathbb{N}}$ is ergodic if $\rho < 1$, recurrent if $\rho = 1$, and transient if $\rho > 1$.*

Proof. We apply Foster's criteria (31). Let $\rho = \frac{\lambda\mathbb{E}[T(N)]}{N} = \frac{\lambda\int_0^\infty tdB_N(t)}{N}$

$$x_i = \begin{cases} 0 & \text{if } i < N \\ i + N & \text{otherwise} \end{cases}$$

For $i \leq N$, we clearly have $\sum_{j=0}^{+\infty} Q_{ij}x_j < \infty$. Now, for $i \geq N$

$$\begin{aligned} \sum_{j=0}^{+\infty} Q_{ij}x_j &= \sum_{j=0}^{+\infty}\mu_{j,N}x_{j+i-N} = \int_0^\infty \sum_{j=0}^{+\infty}(j+i)e^{-\lambda t}\frac{(\lambda t)^j}{j!}dB_N(t) \\ &= \lambda\int_0^\infty tdB_N(t) + i = x_i - \varepsilon \end{aligned}$$

with $\varepsilon = N - \lambda\int_0^\infty tdB_N(t)$. If $\rho < 1$, then $\varepsilon > 0$. This inequality is enough to conclude that the chain is ergodic, and that it is recurrent if $\rho = 1$. As far as the last part of the lemma is concerned, we need an extension of one of Foster's criteria:

Lemma 2. *If there exists a bounded non-constant solution of the equations $\sum_{j=0}^{+\infty} Q_{ij}y_j = y_i$ for $i \geq N$, then the chain is transient.*

Let us construct such a solution in the case $\rho > 1$. For $|p| < 1$ and $i \geq N$, we have

$$\sum_{j=0}^{+\infty} Q_{ij}p^j = \sum_{j=0}^{+\infty}\mu_{j,N}p^{j+i-N} = p^{i-N}\int_0^\infty e^{-\lambda t(1-p)}dB_N(t)$$

where we recognize the Laplace-Stieltjes transform of B_N. Hence, if we were to find $0 < p_0 < 1$ solution of $p^N = \int_0^\infty e^{-\lambda t(1-p)} dB_N(t)$, the vector $y_i = Ap_0^i$ would verify the equations of Foster's criterion, and our chain would be transient. Let us define

$$f(p) = p^N - \int_0^\infty e^{-\lambda t(1-p)} dB_N(t)$$

Clearly, $f(0) < 0$, $f(1) = 0$ and $f'(1) = N - \lambda \int_0^\infty t dB_N(t) = N(1-\rho)$. If $\rho > 1$, the derivative is strictly negative. This guarantees the existence of a zero of f in the interval $]0,1[$, and concludes the proof. □

In the BATCH case, with $\mathcal{CN} = (\{T_N, T_E\}, \varnothing)$, we see that $B_n(t)$ is a sum of three Dirac's functions and verifies

$$T(n) = \begin{cases} nT_1 & \text{with probability } d^{n+1} + (1-d)^{n+1} \\ (n-1)T_1 + T_2 & \text{with probability } 1-p \\ (n-2)T_1 + 2T_2 & \text{with probability } p - d^{n+1} - (1-d)^{n+1} \end{cases}$$

where $p = d^2 + (1-d)^2$ represents in this context the probability of the first vehicles of two consecutive batches having the same trajectory.

Since by Lemma 1 the Markov chain embedded in the queuing process is ergodic if and only if $\lambda < \frac{N}{E[T(N)]}$, the capacity of BATCH can be computed explicitly by deriving $E[T(N)]$, which yields:

$$C_B(N) = \frac{N}{(N-1-p)T_1 + (1+p)T_2 + 2(T_1 - T_2)(d^{N+1} + (1-d)^{N+1})} \ .$$

It is easy to verify that

$$\lim_{N \to \infty} C_B(N) = \frac{1}{T_1} \ ,$$

implying asymptotic optimal capacity of algorithm BATCH.

Delay. Delay statistics can be computed by deriving the stationary queue length distribution π, which implies solving equation $\pi = \pi Q$. Solving this equation is feasible if it is assumed that the system can only contain up to a given number of vehicles – an assumption which is reasonable in practical cases. Under this assumption, the transition matrix Q becomes finite and the queue length distribution can be found by numerically solving the equation above. The resulting first and second order moments of the delay for the BATCH strategy are reported in Table 1 of the main text.

1.7.3 Extension to general configuration

While for the simple two-trajectories case (and, more in general, for any intersection with $\mathcal{CN} = (\mathcal{T}, \varnothing)$) the capacity-optimal way to process a batch of n vehicles is obvious, the general setting with a non-empty set of links E in the compatibility network requires more attention. In particular, it is no longer immediate to define an optimal way of processing a batch of n vehicles, so to characterize the optimal service time distribution $B_n(t)$. Since the number of vehicles n comprised in each batch is not large, it is conceivable to use a brute-force approach to find out the best way of processing requests. More generally, for any choice of request processing algorithm, if the distributions $B_n(t)$ of $T(n)$ can be partially known ($n \in \mathcal{N}$), the methods described above are applicable and offer a way to derive bounds on capacity and delays. For instance, $C_{algorithm} \geq \frac{N'}{\mathbb{E}[N']}$ where $N' = \max(\mathcal{N})$.

2 Video legend

The included video reports a side-by-side comparison of how the two systems (slot-based intersection and traffic light) manages incoming traffic. In the side-by-side comparison shown at the end of the video, the number and time of arrival of vehicles is exactly the same, so to better visually highlight the difference between the two systems. Vehicles are colored with a color scale ranging from white (vehicle traveling at free flow speed) to red (vehicle stopped). The graphics on the left hand side of the screen report the average vehicle delay (top) and estimation of vehicle emissions (bottom) for the two systems.

The video has been ideated at MIT Senseable City Lab, and commissioned to Ryan Yeung (www.ryeung.net) for production. The video has been produced using Autodesk Maya.
